# Editorial: Pathoblockers and antivirulence agents of plant-origin for the management of multidrug resistant pathogens

**DOI:** 10.3389/fmicb.2023.1201495

**Published:** 2023-04-25

**Authors:** Francesca Bonvicini, Manuela Mandrone, Sekelwa Cosa

**Affiliations:** ^1^Department of Pharmacy and Biotechnology, Alma Mater Studiorum, University of Bologna, Bologna, Italy; ^2^Division of Microbiology, Department of Biochemistry, Genetics and Microbiology, University of Pretoria, Pretoria, South Africa

**Keywords:** plant secondary metabolites, essential oils (EOs), multidrug resistant (MDR) pathogens, antibiofilm agents, antivirulence agents

Given the huge diversity of flora and ecosystems in the world, plants likely represent significant sources of bioactive compounds and, consequently, they have been extensively explored for new drug discoveries. Among their bioactivities, plant metabolites, which are the most powerful and promising elements of plants, have been shown to possess antimicrobial potential. It is well known that plant extracts act as antibacterial and antifungal agents (Anand et al., 2019; Mandrone et al., 2019; Adeosun et al., 2022), but they are also a valuable source of anti-virulence compounds with unique mechanism of action targeting pathogenicity or virulence. In addition, some phytochemicals, despite not being directly microbial inhibitors by themselves, show antibiotic adjuvant activity or bio-enhancing activity to attain bactericidal synergism (Patil et al., 2011; Dudhatra et al., 2012); moreover, they serve as reducing and stabilizing agents for the effective synthesis of plant-based metal nanoparticles, improving the antibacterial properties of these nanomaterials (Guleria et al., 2022).

All these features make plants and their plant-derived compounds a desirable alternative to the current antibiotics, which are becoming less and less effective in treating bacterial infections due to the incessantly increase of multidrug-resistant (MDR) pathogens. The MDR pathogens include the groups: 1^st^ Priority- *Pseudomonas aeruginosa, Enterobacteriaceae* (*Klebsiella pneumoniae, Escherichia coli, Serratia* spp., *Proteus* spp., and so forth), 2^nd^ Priority- *Neisseria gonorrhoeae*, amongst other and 3^rd^ Priority- *Heamophilus influenza* and *Shigella* spp. These are placed as global priority pathogens as per World Health Organization (World Health Organization, 2017).

The main aim of the Research Topic “*Pathoblockers and antivirulence agents of plant-origin for the management of multidrug resistant pathogens*” was to uncover the role of plant extracts and their selective phytochemicals as potential anti-virulence candidates to prevent and treat bacterial infections. Within this topic, Wang et al. characterized the overall antibacterial effects of the chlorogenic acid (CA), a natural phenolic compound richly found in fruits and vegetable, on clinical isolates of hypervirulent carbapenem-resistant *K. pneumoniae* (hv-CRKP). These strains, being resistant to different antibacterial drugs and carrying virulence genes of varying degrees, pose an emergent threat to public health. These virulent isolates have high adhesion, and protease and capsular polysaccharide levels, and they use quorum sensing (QS) tactics to activate resistance genes, form biofilm and their associated virulence factors. CA proved to be effective in inhibiting the production of the extracellular polysaccharide matrix, cell attachment and reducing virulence factors by interrupting the QS system. Thus, it can be considered a valuable phytochemical with antibacterial potential to reduce the infectious process of selected MDR bacteria.

Today there is absolutely no doubt that microbial cells within the biofilms have an increased antibiotic tolerance and virulence, and that almost more than 75% of human infections are biofilm-related. As bacterial cells can adhere to virtually all biotic or abiotic surfaces, biofilms are associated to various pathological conditions in humans such as cystic fibrosis, colonization of indwelling medical devices and dental plaque formation involved in caries and periodontitis. With regard to biofilm-related infections of the oral cavity, the study by Idir et al. evaluated the antimicrobial and antibiofilm properties of both ethanolic and aqueous extracts of different Algerian medicinal plants. Among the tested ethanolic extracts, *Origanum vulgare* showed strong antibiofilm activity; the extract prevented *in vitro* biofilm formation of a library of dental plaque isolates, and, remarkably, it was able to reduce the adhesion of strains to a hydroxyapatite coated-surface mimicking the oral niche. Fractionation of the extract and subsequent GS-MS analysis identified the thymol as an important active compound, together with other unidentified phytochemicals that synergistically contributed to the activity of the extract.

Among natural plant-derived molecules, essential oils (EOs) have been used for thousands of years as medicines due to their wide spectrum of biological activities such as antimicrobial, anticancer, antioxidant, anti-inflammatory and antidiabetic. Besides these positive characteristics, some EOs have limited applications in pharmaceutical industries for several disadvantages including poor solubility, extreme volatility and sensitivity to light exposure and elevated temperature. Nanotechnology is a solution to preserve the therapeutic efficacy of EOs while minimizing their physicochemical limitations. In particular, emulsions are gaining special attention because they are inexpensive and scalable (De Luca et al., 2021). In this context, Ganić et al. investigated the antibiofilm potential of a cinnamon EO emulsion and compared data with a commercially available cinnamon EO (*Cinnamomun zeylanicum* L.). Authors selected *Acinetobacter baumanni* as ESKAPE pathogen for their investigations. The ESKAPE strains are six nosocomial pathogens (*Enterococcus faecium, Staphylococcus aureus, K. pneumoniae, A. baumannii, Pseudomonas aeruginosa*, and *Enterobacter* species) that exhibit multidrug resistance and high virulence. In particular, infections associated with *A. baumannii* present a serious problem in intensive care units because of the difficulty of its treatment due to biofilm formation. The EO emulsion displayed remarkable activity against planktonic cells as well as bacterial cells embedded in the extracellular matrix. Strong antibiofilm activity was also demonstrated against a mature biofilm, however, at cytotoxic concentrations indicating that the methodology of cinnamon emulsion synthesis should be improved.

Antimicrobial resistance (AMR) is a global multifaced phenomen not only restricted to humans as its impact is also implicated in fish, and animal diseases. As an example, *Aeromonas hydrophila* is regarded as a significant risk factor in freshwater aquaculture systems possibly leading to humans intestinal and extra-intestinal diseases. Chen et al. investigated the effects of glycyrrhetinic acid ß (GA), an oleanane-type triterpene obtained from *Glycyrrhiza glabra*, on *A. hydrophila* isolated from diseased fishes. Even if the phytochemical did not reduce bacterial growth, it down-regulated the mRNA expression of genes involved in the hemolytic activity of *A. hydrophila*. The development of virulent-targeted medicines is imperative in the war against MDR pathogens and in the prevention of biofilm infections. The therapeutic approaches exploiting pathoblockers and antivirulence agents are less likely to cause resistance as bacteria are unable to develop resistance to multiple chemically complex phytochemicals present in the plant extracts.

Overall, the contributing articles of the Research Topic provide scientific evidence of the potential benefits of plant derived compounds and the importance of the development of new therapeutic agents affecting key events in the pathogenic process rather than killing the microorganism itself.

## Author contributions

FB: writing—original draft preparation. MM and SC: writing—review and editing. All authors contributed to the article and approved the submitted version.
